# Homocysteine aggravates ROS-induced depression of transmitter release from motor nerve terminals: potential mechanism of peripheral impairment in motor neuron diseases associated with hyperhomocysteinemia

**DOI:** 10.3389/fncel.2015.00391

**Published:** 2015-10-06

**Authors:** Ellya Bukharaeva, Anastasia Shakirzyanova, Venera Khuzakhmetova, Guzel Sitdikova, Rashid Giniatullin

**Affiliations:** ^1^Department of Physiology, Kazan Federal UniversityKazan, Russia; ^2^A. I. Virtanen Institute for Molecular Sciences, Department of Neurobiology, University of Eastern FinlandKuopio, Finland; ^3^Kazan Institute of Biochemistry and BiophysicsKazan, Russia

**Keywords:** amyotrophic lateral sclerosis, neuromuscular junction, homocysteine, oxidative stress, NMDA receptors

## Abstract

Homocysteine (HCY) is a pro-inflammatory sulphur-containing redox active endogenous amino acid, which concentration increases in neurodegenerative disorders including amyotrophic lateral sclerosis (ALS). A widely held view suggests that HCY could contribute to neurodegeneration via promotion of oxidative stress. However, the action of HCY on motor nerve terminals has not been investigated so far. We previously reported that oxidative stress inhibited synaptic transmission at the neuromuscular junction, targeting primarily the motor nerve terminals. In the current study, we investigated the effect of HCY on oxidative stress-induced impairment of transmitter release at the mouse diaphragm muscle. The mild oxidant H_2_O_2_ decreased the intensity of spontaneous quantum release from nerve terminals (measured as the frequency of miniature endplate potentials, MEPPs) without changes in the amplitude of MEPPs, indicating a presynaptic effect. Pre-treatment with HCY for 2 h only slightly affected both amplitude and frequency of MEPPs but increased the inhibitory potency of H_2_O_2_ almost two fold. As HCY can activate certain subtypes of glutamate N-methyl D-aspartate (NMDA) receptors we tested the role of NMDA receptors in the sensitizing action of HCY. Remarkably, the selective blocker of NMDA receptors, AP-5 completely removed the sensitizing effect of HCY on the H_2_O_2_-induced presynaptic depressant effect. Thus, at the mammalian neuromuscular junction HCY largely increases the inhibitory effect of oxidative stress on transmitter release, via NMDA receptors activation. This combined effect of HCY and local oxidative stress can specifically contribute to the damage of presynaptic terminals in neurodegenerative motoneuron diseases, including ALS.

## Introduction

Homocysteine (HCY, 2-amino-4-sulfanylbutanoic acid) is a sulphur-containing endogenous amino acid, which is produced in the methylation cycle of protein metabolism and involved in maintaining the cells redox balance. An elevated plasma level of HCY (termed hyperhomocysteinemia, hHCY) markedly decreases cell viability (Kolling et al., 2013). Extremely high HCY levels (up to 200 μM) has been found in some patients with disrupted HCY metabolism, and is believed to be at the root of certain vascular disorders including stroke and coronary occlusions (Kang et al., 1992; Stanger et al., 2009). According to a number of studies, abnormally elevated levels of HCY in plasma and cerebrospinal fluids correlate with amyotrophic lateral sclerosis (ALS), a motor neuronal disease that causes muscle degeneration (Zoccolella et al., 2008, 2010; Levin et al., 2010; Valentino et al., 2010; Veeranki and Tyagi, 2013). Inherited deficiency of the enzyme methylenetetrahydrofolate reductase (MTHFR), transforming HCY into methionine through remethylation, is associated with severe muscular hypotonia (Huemer et al., 2015). There is conflicting evidence about the role of the frequent MTHFR C677T polymorphisms as risk factors for ALS (Kühnlein et al., 2011; Ricci et al., 2012; Sazci et al., 2012). The variant MTHFR C677T, known to be associated with increased levels of HCY (Kang et al., 1988; Li et al., 2003) is more frequent among ALS patients (Kühnlein et al., 2011), however an Italian population study found no association (Ricci et al., 2012). Apart from a direct link between MTHFR and ALS, there are other possible mechanisms how the increasing level of HCY due to aging or improper diet (Zhang et al., 2008; Song et al., 2013) can speed up the development of disease in patients with ALS.

Recently, a link of the other Ca^2+^-dependent enzyme transglutaminase 2 with several neurodegenerative diseases including ALS has been proposed in a study showing HCY-induced activation of THP-1 monocytes associated with oxidative stress (Gurrò et al., 2015).

The role of HCY in ALS is supported by several studies showing the beneficial effects of folate or vitamin B12/B6 treatments (Zhang et al., 2008; Song et al., 2013), which are well known most efficient approaches to lower the level of endogenous HCY. In line, a large-scale human trial on the action of methylcobalamin in ALS patients is currently ongoing (Ikeda et al., 2015).

According to the “dying-back” hypothesis of ALS (Moloney et al., 2014; Pollari et al., 2014) the dysfunction of nerve terminals precedes pathological neurodegenerative changes in a motoneuron. One of the commonly reported mechanisms of HCY action is the enhanced production of reactive oxygen species (ROS; Loureiro et al., 2010; Lee et al., 2013; Veeranki and Tyagi, 2013). In the neuromuscular junction oxidative stress induced by hydrogen peroxide (H_2_O_2_) inhibits synaptic transmission at the presynaptic site (Giniatullin and Giniatullin, 2003; Giniatullin et al., 2006). The role of oxidative stress in degeneration of the neuromuscular junction in ALS was reviewed by us recently in the frame of this research topic (Pollari et al., 2014). However, despite the recent reports that hHCY is associated with impaired muscle functions (Swart et al., 2013; de Jager, 2014) the action of HCY on neuromuscular transmission and its role in the oxidative damage of motor nerve terminals has not been investigated so far.

Thus, the first aim of the current study was to test the ability of HCY to promote the depressant action of ROS on synaptic transmission using isolated neuromuscular junctions.

Several independent observations are pointing towards a potential correlation between enhanced glutamate receptor signaling and ALS progression. In the nervous system, extracellular HCY is able to stimulate the glutamate N-methyl D-aspartate (NMDA) receptor (Lipton et al., 1997; Poddar and Paul, 2013; Abushik et al., 2014). Homocysteinic acid, an analog of HCY with potent agonist activity on NMDA receptors triggers calcium accumulation in motor neurons (Adalbert et al., 2002). Increased glutamate levels were found in blood plasma of patients with ALS (Cecchi et al., 2014). A presynaptic glutamate release inhibitor Riluzole applied to ALS patients as a medical treatment extends life by 3–5 months (Miller et al., 2012) suggesting a role of glutamate receptors in the progression of this disease.

At the mammalian neuromuscular junction both NR1 and NR2A subunits of NMDA receptor have been found (Mays et al., 2009). In rats, the inhibition of NMDA receptors suppressed contractions of the diaphragm muscle, without changing acetylcholine (ACh) signaling (Koyuncuoğlu et al., 1998). Hyperactivation of NMDA receptors is often associated with oxidative stress (Lee et al., 2013), calcium overload and ROS generation (Ratan and Baraban, 1995). Thus, the second aim of this study was to test the involvement of muscle NMDA receptors in synaptic action of HCY and ROS. For this aim, the effect of HCY and H_2_O_2_ on transmitter release was investigated at the mouse neuromuscular junctions pre-treated with the selective blocker of NMDA receptors.

## Materials and Methods

### Preparation and Solutions

Experiments were performed on isolated mice phrenic nerve—diaphragm preparations kept at room (22–23°C) temperature. Mice (BALB/c strain) of both sexes of 20–25 g body weight were euthanized in accordance with regulations of the European Community Council Directive (September 22, 2010; 2010/63/EEC). Animal experiments were approved by the Ethical Committee of the University of Eastern Finland. Preparations were placed in a small 3.5-ml volume chamber and continuously superfused with gassed (95% O_2_/5% CO_2_) physiological solution containing (in mM): NaCl 120; KCl 5; CaCl_2_ 2; NaHCO_3_24; NaHPO_4_ 1; MgCl_2_ 1; glucose 11, at pH 7.3–7.4. All reagents were purchased from Sigma-Aldrich (St. Louis, MO, USA) and were dissolved directly before the use. We used D, L-HCY with expected ~50% of the natural active L-form of HCY (Lipton et al., 1997). Drugs were applied via bath perfusion (rate ~ 2 ml/min).

### Electrophysiology

Miniature end-plate potentials (MEPPs) recorded by microelectrodes (resistance 5–8 MΩ; filled with 3 mM KCl) as shown in details in Naumenko et al. (2011). In brief, MEPPs were acquired using the data acquisition board NI PCI6221 (National Instruments, Austin, TX, USA), visualized with WinEDR V3.2.7 software (Strathclyde University, UK), digitally stored at 50 kHz. Off-line amplitudes and interevent intervals of MEPPs were analyzed with the WinEDR V3.2.7 and ClampFit V10.2.0.14 (Molecular Devices, Sunnyvale, CA, USA) software. Subsequent statistical analysis was made using the Origin graphic software (Origin Lab Corp, Northampton, MA, USA). MEPPs were collected during 3 min from each of 8–10 synapses at the same muscle preparation to calculate mean values of amplitudes and frequencies in control solution and after 30–35 min application of 300 μM H_2_O_2_. Muscle fibers with a resting membrane potential less negative than −60 mV were rejected from further analysis.

### Statistical Analysis

In each experimental series H_2_O_2_ effect was counted by comparing the mean MEPP frequencies and amplitudes before and after ROS application (30–35 min). Mean values before H_2_O_2_ were taken as 100%. The data are presented as the mean ± SEM (*n* = number of synapses with indication of number of animals used). Statistical significance was assessed by using Student’s *t*-test (for parametric data) or Mann-Whitney test (for nonparametric data). Differences were considered significant when *p* < 0.05.

## Results

**Figure 1:**
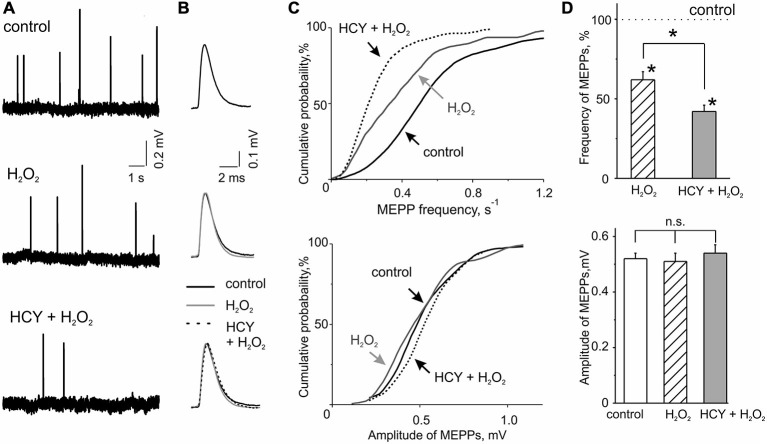
**H_2_O_2_ inhibitory action on spontaneous acetylcholine (ACh) release from nerve endings at intact and homocysteine (HCY) pre-treated mice diaphragms. (A)** Representative traces of MEPPs in control, after 300 μM H_2_O_2_ application at intact and pre-treated by HCY (500 μM, 2 h) diaphragm muscles. **(B)** MEPP shapes before, 30–35 min after application of 300 μM H_2_O_2_ in intact and pre-incubated with HCY (500 μM, for 2 h) preparations. Average of 25–30 MEPPs. **(C)** Cumulative curves of MEPP frequencies and amplitudes in control and after H_2_O_2_ application at intact and HCY pre-treated muscles. **(D)** The top panel—mean MEPP frequencies in the presence of 300 μM H_2_O_2_ at intact (*n* = 47 synapses/6 mice), and HCY-pre-incubated (*n* = 55 synapses/7 mice) muscles compared to the frequency level before H_2_O_2_ application (taken as 100%), ^*^p < 0.05; the bottom panel—mean MEPP amplitudes (mV) before, 30–35 min after application of 300 μM H_2_O_2_ in intact (*n* = 47 synapses/6 mice) and pre-incubated with HCY (500 μM, for 2 h; *n* = 55 synapses/7 mice) preparations, ns = non significant.

We first tested the action of acute oxidative stress on transmitter release in control conditions. MEPPs representing spontaneous release of ACh quanta from nerve terminals occurred at a mean frequency of 0.60 ± 0.05 s^−1^ and an amplitude of 0.52 ± 0.02 mV (*n* = 46 synapses/6 mice). These quantal events are classical readouts of the functional state of the motor synapse when the frequency of MEPPs reflects the presynaptic function whereas the amplitude of MEPPs characterizes the functional state of the postsynaptic membrane (Fatt and Katz, 1952). To model acute oxidative stress we used the mild oxidant H_2_O_2_ in concentration of 300 μM. Based on our previous experience (Giniatullin et al., 2006) we considered this concentration efficient and safe. H_2_O_2_ applied via bath perfusion significantly reduced the frequency of MEPPs (Figures 1A,C,D). After 30–35 min exposure to H_2_O_2_ the mean frequency of MEPPs decreased by 32.3 ± 8.6% (*n* = 47 synapses/6 mice, *p* < 0.05 by Mann-Whitney test) regarding control untreated preparation. No changes were observed in MEPP amplitudes (47 synapses/6 mice, *p* > 0.05; Figures 1A–D) indicating a pure presynaptic effect of H_2_O_2_.

The temporal characteristics of MEPP were not changed by H_2_O_2_. Thus, the rise-time in control was 0.20 ± 0.01 ms (*n* = 47 synapses/6 mice) and the decay time constant of MEPP was 3.18 ± 0.28 ms (*n* = 44 synapses/5 mice). After H_2_O_2_ treatment these parameters remained unchanged (95 ± 7% and 94 ± 8% from control values, respectively, *n* = 44 synapses/5 mice,*p* > 0.05).

Next, we tested the action of HCY on spontaneous ACh release and whether this exposure changed the inhibitory effect of H_2_O_2_. As we intended to model naturally long-lasting hHCY in the short time window we selected the relatively large concentration of HCY of 500 μM which nevertheless corresponds the severe hHCY in humans (Kang et al., 1992; Stanger et al., 2009). Notably, as we used the racemic D, L-form of HCY, the effective concentration of the naturally occurring L-form was actually only half of the total (Lipton et al., 1997). Nevertheless, after incubation of the neuromuscular preparations for 2 h in solution containing 500 μM HCY, the mean frequency of MEPPs was the same as in untreated preparations (+5.8 ± 9.0% change comparing with untreated samples; *n* = 56 synapses/6 mice). No changes were observed in the amplitudes of MEPPs (Figures 1A–D) and in the time-course of MEPPs (Figure 1B) after HCY exposure (rise-time = 94 ± 5% and the decay time constant = 95 ± 7%, *p* > 0.05, *n* = 49 synapses/6 mice).

However, after HCY pre-treatment (500 μM, 2 h) the inhibitory potency of H_2_O_2_ was largely increased. MEPPs frequency decreased by 60.0 ± 3.6% from the level in the presence of HCY before H_2_O_2_ application (*n* = 55 synapses/7 mice, *p* < 0.05; Figures 1A–D). Thus, the depressant effect of H_2_O_2_ on spontaneous ACh release was significantly (*p* < 0.05 by Mann-Whitney test) more (almost two times) efficient after muscles exposure to HCY compared to the action of H_2_O_2_ alone. No changes were observed in the amplitudes of MEPPs (Figures 1A–D). Thus, HCY largely amplified the impairment of motor nerve terminals induced by acute oxidative stress.

**Figure 2:**
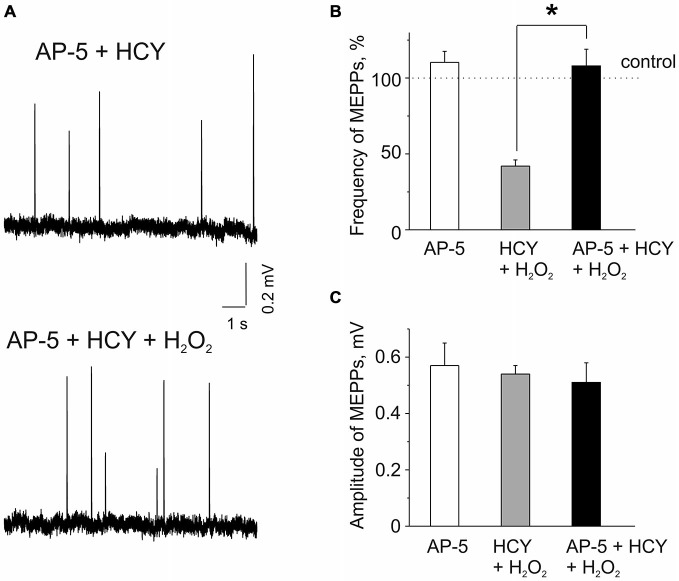
**Effects of AP-5 and the action of H_2_O_2_ on MEPPs after pre-incubation of neuromuscular junctions in HCY or in the mixture of AP-5 and HCY. (A)** MEPPs (top) in muscles pre-incubated in the selective blocker of N-methyl D-aspartate (NMDA) receptors AP-5 (50 μM, for 20 min), following incubation in a mixture of AP-5 and 500 μM HCY for 2 h, and MEPPs (bottom) after application of 300 μM H_2_O_2_ in the presence of AP-5 + HCY. **(B)** Effects of 50 μM AP-5 alone and the action of H_2_O_2_ on MEPP frequencies in muscles pre-incubated for 2 h in 500 μM HCY and in the mixture of AP-5 and HCY (*n* = 53 synapses/6 mice, *p* > 0.05). MEPPs frequency level in untreated muscle (for AP-5 alone) or the levels in HCY and in AP-5 + HCY before H_2_O_2_ application was set as 100%. Notice that AP-5 largely reduced the inhibitory effect of H_2_O_2_ in the presence of HCY, ^*^p < 0.05. **(C)** MEPP amplitudes in AP-5 and before and after 30–35 min application of 300 μM H_2_O_2_ to diaphragm muscles pre-incubated in 500 μM HCY and in mixture of 50 μM AP-5 and 500 μM HCY (*n* = 53 synapses/6 mice), ns = non significant.

Next, we explored potential mechanisms underlying the sensitizing effect of HCY. As we (Abushik et al., 2014) and others (Lipton et al., 1997; Poddar and Paul, 2013) showed that HCY can act *via* activation of NMDA receptors we next tested the role of these receptors in the sensitizing action of HCY. Several previous reports indicated the presence of NMDA receptors at the neuromuscular junction (Grozdanovic and Gossrau, 1998; Mays et al., 2009; Malomouzh et al., 2011; Walder et al., 2013). In these experiments we applied H_2_O_2_ after 20 min pre-incubation with the selective NMDA receptor blocker AP-5 (50 μM, Abushik et al., 2014), following by incubation of muscles with a mixture of 50 μM AP-5 and 500 μM HCY for 2 h. Remarkably, H_2_O_2_ (300 μM) applied after muscle incubation in the mixture of AP-5 plus HCY did not cause the depressant action on spontaneous ACh release from nerve endings (Figures 2A,B). Thus, MEPPs frequency in 30–35 min of H_2_O_2_ exposure was even by 8.8 ± 11.1% higher than control level after exposure to AP-5 + HCY (*n* = 53 synapses/6 mice, *p* > 0.05). However, a similar insignificant trend (10 ± 7% change, *n* = 48 synapses/5 mice, *P* = 0.85) was also observed when AP-5 was applied to the muscle alone (Figure 2B). MEPP amplitudes were not significantly changed by H_2_O_2_ applied after pre-exposure to AP-5 + HCY (Figures 2A,C). Likewise, MEPPs kinetics was not significantly changed (rise-time = 96 ± 6% and the decay time constant = 94 ± 8% of control values in AP-5 + HCY, *n* = 53 synapses/6 mice, *p* > 0.05).

The results of these experiments indicated NMDA receptor-mediated mechanism of sensitizing action of HCY.

## Discussion

The main finding of our study is that the redox active HCY increases the vulnerability of the mammalian neuromuscular junction to the inhibitory action of ROS via a glutamatergic mechanism. This finding uncovers a novel plausible mechanism for peripheral synaptic impairments, which may be relevant to motor neuron diseases such as ALS, as these disorders can be associated with hHCY and with oxidative stress. By modeling these conditions, our study suggests a new explanation for why motoneurons (and their peripheral parts) are more vulnerable to ALS associated with hHCY than other type of neurons.

**Figure 3:**
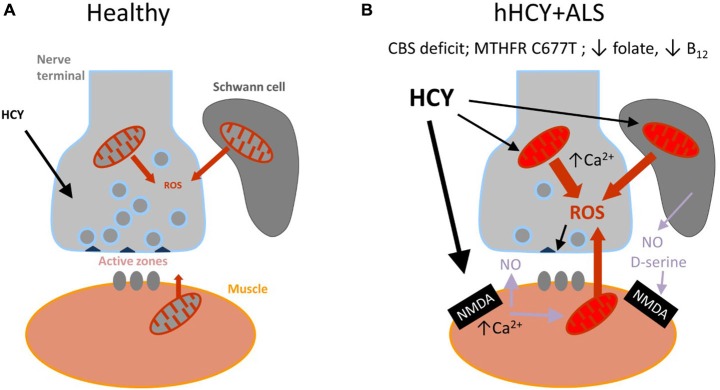
**Schematic presentation of the model proposed for the combined damaging action of hyperhomocysteinemia (hHCY) and oxidative stress to motor nerve terminals. (A)** Healthy (normal low level of HCY) neuromuscular junction comprising motor nerve terminals, surrounding Schwann cells and muscles fibers. reactive oxygen species (ROS) generated via mitochondria are mostly neutralized by the endogenous antioxidant mechanisms. All this provided high level of transmitter release from presynaptic active zones. **(B)** Compromised motor nerve terminals in amyotrophic lateral sclerosis (ALS) associated with hHCY due to polymorphism of MTHFR, deficiency of cystathionine beta-synthase (CBS) or improper diet (low folate or B12). Notice the reduced number of active zone and synaptic vesicles and compromised mitochondria at different levels (muscle, nerve and Schwann cells) generating increased level of ROS. Activation of postsynaptic NMDA receptors by high HCY assisted by the co-agonist D-serine released from glial cells can lead to Ca^2+^-dependent induction of NO, known as a strong presynaptic inhibitor by itself and partner in generation of toxic ONOO^−^.

There is strong evidence of the development of oxidative stress and of the detrimental role of ROS in ALS (reviewed in Pollari et al., 2014). At the neuromuscular junction, ROS are primarily generated at postsynaptic sites during intense muscle activity (Jackson, 2008) and can be induced via presynaptic mitochondria activity (David and Barrett, 2003; see also Figure 3). Dysfunction of mitochondria and Ca^2+^ dysregulation contribute to the muscle denervation and motor neuron death that occur in mouse models of ALS (Jaiswal, 2013; Barrett et al., 2014).

The presynaptic transmitter releasing machinery is the most redox-sensitive part of the motor synapse (Giniatullin and Giniatullin, 2003; Giniatullin et al., 2006) and such a mechanism probably contributes to the muscle fatigue during the development of ALS (Pollari et al., 2014).

Despite the recent report that hHCY is associated with impaired muscle functions (Swart et al., 2013; de Jager, 2014) the action of HCY has not yet been studied directly at the neuromuscular junction. However, the role of HCY in ALS where the impairment of the neuromuscular transmission is one of the early and leading symptoms of the disease is consistently discussed. Thus, many studies reported the high level of HCY in ALS (Zoccolella et al., 2008, 2010; Levin et al., 2010; Valentino et al., 2010; Veeranki and Tyagi, 2013). In particular, high levels of HCY in both plasma and cerebrospinal fluids were found to correlate with ALS muscle degeneration (Zoccolella et al., 2008; Valentino et al., 2010). However, conflicting data should also be mentioned. Thus, while some authors found a link between MTHFR C677T polymorphisms as risk factors for ALS (Kühnlein et al., 2011; Sazci et al., 2012) others (Ricci et al., 2012) did not detect a direct association between the two.

Although a causal link remains disputable, the *simultaneous presence* of hHCY and ALS associated oxidative stress in nerve terminals could initiate the mechanism of presynaptic depression observed in the current study. According to this view there is a “two keys mechanism” when combined HCY and ROS synergistically impair the presynaptic compartment of the neuromuscular junction (Figure 3). The attractiveness of this hypothesis is that it integrates all of the three potential partners of the neuromuscular junction—muscle fibers, Schwann cells and nerve terminal which all could be sources of ROS (Figure 3).

HCY induced exacerbation of ALS is supported by clinical evidence of the favorable effect of HCY-lowering folate and B12 supplements (Zhang et al., 2008; Song et al., 2013; Ikeda et al., 2015). One could suggest that the measurement of endogenous level of HCY in ALS patients should be monitored to determine the best type of treatments (and perhaps prophylaxis). Increase in HCY levels by low plasma folate or improper nutrition (Kang et al., 1992) might be an additional overlapping condition aggravating the development of ALS. Therefore, even though unable to cure ALS alone, the folic acid therapy could be an important component of a more complex disease therapy. Another important implication of our results is that patients with ALS should avoid nitrous oxide for anaesthesia as the latter could induce an acute burst of HCY (Asghar and Ali, 2012; Morris et al., 2015).

Interestingly, increased total HCY levels can be detected in a variety of neurological diseases such as stroke (Kang et al., 1992), Alzheimer’s disease and other types of dementia (Isobe et al., 2005; de Jager, 2014), psychiatric disorders or infections such sepsis (Ploder et al., 2010) without reported signs of ALS symptomatology. This raises a question: why would ALS symptomatology develop only in this specific group of patients? To answer to this question we propose a working hypothesis based on the main finding of the current study: the interaction between ROS and HCY, that whereas hHCY is not specific to ALS (being observed in other above-mentioned diseases) *the coincidence* of hHCY and oxidative damage locally in the peripheral part of the motoneuron *is specific* to ALS. In addition, the level of HCY is almost 10 times higher in blood than in the CSF (Valentino et al., 2010) suggesting a higher probability of HCY-mediated toxicity in peripheral tissues such as nerve endings in the skeletal muscle. Thus, our hypothesis can provide a novel explanation of why peripheral endings of the motoneuron are more vulnerable to ALS than central neurons affected by other brain diseases.

In the current study, in order to model the detrimental effects of HCY in a short time frame we used high concentrations of the D-L form of HCY. In fact, the actual concentration of the active L-form of HCY (Lipton et al., 1997) in our experiments is at least two times lower (~250 μM) which is close to what is observed in severe hHCY (Kang et al., 1992; Stanger et al., 2009). Notably, even at such high concentrations HCY did not significantly affect neuromuscular transmission parameters *per se*. The accuracy of measurement of the fraction of total and free HCY in pathological states is still a matter of debate (Adam et al., 2013). It is also worth noting that, in addition to HCY, a related endogenous compound homocystine is present in the blood (Zhang et al., 2014), and its molecular targets deserve future studies.

The main finding of the current study was that the exposure to HCY largely promoted the presynaptic inhibitory action of H_2_O_2_. Moreover, in our study we provide a mechanistical explanation for this phenomenon identifying the key role of glutamate NMDA receptors in the sensitizing effect of HCY. Indeed, the selective blocker of NMDA receptors AP-5 completely prevented the additive effect of HCY on the inhibition of transmitter release by H_2_O_2_. Our finding is consistent with previous studies showing that HCY induces excitotoxic effects mainly in cells expressing NMDA receptors (Boldyrev et al., 2013). Activation of NMDA receptors by HCY results in an increase of cytoplasmic Ca^2+^ and ROS accumulation (Loureiro et al., 2010; Leal and Gomes, 2015) which all are likely happening in skeletal muscle during hHCY (Figure 3).

Glutamate ionotropic receptors are located at vertebrate neuromuscular junctions (Grozdanovic and Gossrau, 1998). Knockouts of NMDA receptor subunits in mice induces phenotypes which are similar to those typically associated with neuromuscular disorders (Single et al., 2000; Meng et al., 2003). The presence of AP-5 sensitive NMDA receptors in the muscle has been shown in several previous studies involving immunolabeling and electrophysiological recordings (Mays et al., 2009; Malomouzh et al., 2011; Walder et al., 2013). However, in contrast to the high diversity of glutamate receptor subunits observed in the CNS, only a limited number of glutamate receptor subunit types is expressed in the neuromuscular junction (Mays et al., 2009). As we did not see changes in the amplitude of MEPPs, we suggest only limited and probably diffuse expression of postsynaptic NMDA receptors in skeletal muscle.

Downstream NMDA receptor signaling pathway could involve the highly diffusible messenger NO (Figure 3). It is known that in neurons, glutamate, acting on post-synaptic NMDA receptors can induce NO release as nitric oxide synthase is a part of the NMDA/NOS complex (Courtney et al., 2014). Consistent with this, HCY neurotoxicity could be due to the release of NO (Stamler et al., 1993) which is a potent inhibitor of ACh release from motor nerve endings (Giniatullin et al., 2005; Valiullina and Sitdikova, 2012). Early studies indicate that the activation of muscle NMDA receptors by bath-applied glutamate increases synthesis of NO in muscle fibers and NO acts in a retrograde manner on motor nerve terminals to inhibit non-quantal release of ACh (Malomouzh et al., 2003).

NO can also cooperate with superoxide to produce the strong oxidant, peroxynitrite, which is considered as a candidate inducer of cell death in ALS (Urushitani and Shimohama, 2001). Whereas the involvement of NO in HCY induced sensitizing effects require further investigations, especially taking into account potential species-dependent action of NMDA/NO mediated signaling which could explain the fact that the retrograde presynaptic inhibition by ROS pre-conditioned by HCY was prevented by the NMDA antagonist AP-5.

Schwann cells surrounding nerve terminals could be additional contributors to the impaired presynaptic function (Pollari et al., 2014) via release of gliotransmitters (Araque et al., 2014) including D-serine (Wu et al., 2005; see also Figure 3). Glial cells D-serine like glycine can potentiate the neurotoxicity of HCY via the glycine binging site of NNDA receptor (Wolosker, 2007; McCully, 2009; see also Figure 3B). In line with this, D-serine can contribute to the pathophysiology of familial ALS associated with the R199W mutation in D-amino acid oxidase (Paul and de Belleroche, 2015).

In summary, we show here that motor nerve terminals are highly vulnerable to the combined action of HCY and oxidative stress, both of which can be present in ALS and likely contribute together to the presynaptic impairment of the neuromuscular function. Thus, we propose a model, which may be relevant for ALS and raises a number of important questions with significant translational impact, which could be investigated further in animal ALS models and in future clinical studies.

## Funding

This project was supported by the Russian Scientific Fund (Grant No 14-15-00618).

## Conflict of Interest Statement

The authors declare that the research was conducted in the absence of any commercial or financial relationships that could be construed as a potential conflict of interest.
